# The effects of ketogenic and chitosan-based diets on submandibular salivary gland in rat model: a comparative histological study

**DOI:** 10.1186/s12903-024-03885-8

**Published:** 2024-01-31

**Authors:** Mahmoud Mohamed Aboulfotoh

**Affiliations:** Oral Biology Department, Faculty of Oral and Dental Medicine, Delta University for Science and Technology, Gamasa, Egypt

**Keywords:** Amyloidosis, Smooth muscle actin, Keto diet, Chitosan-based diet, Submandibular salivary gland

## Abstract

**Objective:**

This study was carried out in the submandibular salivary glands (SSGs) of rats to demonstrate the effect of a ketogenic diet (KD) in comparison with dietary chitosan supplementation.

**Method:**

Eighteen albino rats were randomly divided into three equal groups of six animals each. Rats in Group I were fed a balanced diet and considered controls. Meanwhile, those of Groups II and III were fed a KD, a balanced diet with high molecular weight chitosan, respectively. After 45 days, rats were euthanized, and the SSGs were dissected carefully for staining with hematoxylin and eosin (H&E), alpha-smooth muscle actin (α-SMA) immunohistochemical staining, and Congo red special stain. Quantitative data from α-SMA staining and Congo red staining were statistically analyzed using one-way ANOVA followed by Tukey’s multiple comparisons post hoc test.

**Results:**

Regarding Congo red and α-SMA staining, one-way ANOVA revealed a significant difference between the three groups. For α-SMA staining and Congo red staining, Group II had the highest mean values of 91.41 ± 3.30 and 68.10 ± 5.04, respectively, while Group I had the lowest values of 56.13 ± 3.96 and 16.87 ± 2.19, respectively. Group III had mean values of 60.70 ± 3.55 for α-SMA and 19.50 ± 1.78 for Congo red. Tukey’s multiple comparisons post hoc test revealed significant differences between groups I & II and between groups II & III (*P* < 0.05). Meanwhile, there was a nonsignificant difference between groups I and III (*P* > 0.05).

**Conclusion:**

A KD has a deleterious effect on rats’ SSG whatever the test we used, and dietary chitosan supplementation ameliorates these damaging effects.

## Introduction

In the mouth, salivary glands (SSGs) play a crucial role; they produce a translucent, alkaline liquid that is secreted in the oral cavity for several purposes. The submandibular, sublingual, and parotid glands are the three primary salivary glands that produce 95% of the saliva, with more minor salivary glands producing the remaining 5%. An acinar cell in the salivary gland secretes saliva. Mucous acinar cells and serous acinar cells produce saliva. Thick mucus is made in the vacuoles of mucous cells, while water and enzymes are made in the vacuoles of serous cells [1].

The role of the salivary gland is to produce saliva which is considered a highly complex mixture of water with organic and non-organic substances. It is important for oral hygiene as it lubricates the oral cavity, aids in mastication and swallowing, and finally protects the teeth [2]. Producing saliva serves multiple functions, including moisturizing the mucosal surface, adjusting the pH level to become more alkaline, managing the microbiota, initiating food digestion, and creating the food bolus [3]. There are two categories of saliva: serous and mucosal. The former primarily comprises enzymes and fluids, focusing on the amylase enzyme to break down carbohydrates [3, 4]. The second type, mucosal saliva, is identified by a high concentration of mucin, which causes it to appear thick and sticky. This aids in binding the partially chewed food particles together before swallowing them [5]. If the structure and integrity of the glands are altered, it affects the function and activity of the glands, as well as the flow and composition of saliva [6]. As a result, altered gland morphology directly impacts blood glucose levels, affecting overall health [7].

The ketogenic diet (KD), a daily dietary plan, relies on increased fat intake, moderate protein consumption, and reduced carbohydrates. It has proven to be an effective therapeutic approach for conditions with metabolic factors, effectively reducing seizures in persistent cases of childhood epilepsy [8], reducing blood glucose levels in type 2 diabetes mellitus [9], and assisting in weight loss [10]. However, it should be noted that in the liver, regardless of weight loss, a KD can induce liver fibrosis and nonalcoholic steatohepatitis by increasing liver cholesterol, interleukin-6, and phospho-Jun N-terminal kinase. Additionally, it can exacerbate diet-induced glucose intolerance and hepatic insulin resistance compared to a high-fat diet [11].

While the brain typically relies on carbohydrates as its main energy source, it is less efficient at metabolizing lipids, despite adipose tissue serving as a larger energy store than muscle and liver glycogen. However, during starvation, the liver utilizes lipids to generate ketone bodies, which act as an alternative fuel for the brain. The production of ketone bodies, including D-b-hydroxybutyrate and acetoacetate, gradually increases due to macronutrient availability (low glucose and high free fatty acids) and hormonal signaling (low insulin, high glucagon, and cortisol). Although the body continuously produces ketone bodies under normal physiological conditions, their blood concentrations rise during, following KD, or after prolonged exercise [12].

Chitosan, a dietary fiber derived from shrimp and lobster shells, is a novel feed additive that is not widely utilized. It has been found to exhibit several beneficial biological properties, including antibacterial, anticancer, antioxidant, and hepatoprotective effects. Adding chitosan and its derivatives to diets could aid in reducing cholesterol and glucose levels, making it a viable option for promoting weight loss [13, 14]. Chitosan helps the salivary glands make new tissue structures by controlling the components of the basement membrane. In an environment with chitosan, basement membrane components were more active in space and time [15]. A primary function of chitosan is regulating basement membrane dynamics by enhancing Col IV expression and assisting dystroglycan localization, which is necessary for submandibular gland formation [16]. By interacting with glycosaminoglycans (GAGs), which are extracellular matrix constituents, the existing or changed functional groups of chitosan facilitate the regulation of signals and factors needed for tissue regeneration. Growth factors, nucleic acids, and cytokines are negatively charged compounds that protonated chitosan binds to both in vivo and in vitro. As a result, chitosan enables the integration of numerous bioactive substances that may promote the growth of certain cells or cause stem cells to differentiate into functional differentiated cells, which is frequently a requirement for regeneration [17]. The potential of chitosan to support growth and differentiation in salivary gland epithelium has also been investigated [18]. Yang et al. (2012) [15] revealed that submandibular gland explants exhibited enhanced growth when cultured with chitosan: FGF7. This finding suggests that chitosan could function as a growth stimulator for factors derived from the mesenchyme. This study investigated the effect of KD and dietary chitosan supplementation on rat submandibular salivary glands.

## Materials and methods

### Sample size calculation

The sample size was calculated using G power version 3.1 statistical software (Franz Faul). Fixed-effects, omnibus, 1-way analysis of variance (ANOVA) was performed to compute the sample size given α, power, and effect size. The input parameters were an α error probability of 0.05, an effect size of 6.829, a power of 0.80, and 3 groups. The findings indicated a minimum sample size of 18 rats.

### Materials and chemicals

Corn oil and animal fats (beef tallow) were obtained from local markets in Tanta, Egypt. Low-MW (91 kDa) chitosan prepared from crab shell chitin was purchased from Almolok Chemicals Company, Cairo, Egypt. According to company guidelines, chitosan extraction is done in three steps which are demineralization, deproteination, and deacetylation [19]. Casein, cellulose, vitamin, and mineral mixtures were obtained from Al- Al-Gomhuria Company, Cairo, Egypt. Corn starch was obtained from a local market in Tanta, Egypt.

### Diet preparation

The basal diet of the rats was prepared following the laboratory animal diet guidelines as previously described [20]. The ketogenic diet was prepared according to [21]. The chitosan diet was prepared as a basal diet + 5% low-MW (91 kDa) chitosan according to [22]. The diet formulations are shown in Table 1.

**Table 1:** Compositions of the experimental diets

Ingredients	(g/kg Diet)		
	Control (Basel diet)	Ketogenic diet	Chitosan + Basel diet
Corn oil Total fat beef tallow	40	30	40
	-	570	-
Casein (> 85% protein)	140.0	250	140.0
Mineral mix	35.0	35.0	35.0
Vitamin mix	10.0	10.0	10.0
L- cystine	1.8	1.8	1.8
Choline bitartrate	2.5	2.5	2.5
Fiber	50.0	50	50.0
Carbohydrate	720.7	50.7	670.7
Chitosan	-	-	50

### Experimental protocol

This experiment was performed in the animal house of Delta University for Science and Technology. The experiment was done under the guidelines of ARRIVE (Animal Research: Reporting of In Vivo Experiments) and approval was obtained from the Ethical Committee, Faculty of Dentistry, Delta University for Science and Technology (No. FODMRC-2022-00102). In this study, eighteen albino rats were used with a weight of 180 ± 20 gm and 12–14 weeks purchased from the Vaccine and Immunity Organization, Ministry of Health, Egypt. Under standard conditions, the animals were housed in well-aerated and suitable size cages (12/12 light/dark cycle, 23 ± 2 °C temperature, and 60% humidity old. They were fed a basal diet ad libitum and had free access to water. The animals were acclimatized for one week; then randomized divide into three groups for six animals for each group and only three rats per cage. group I were fed a basal diet. Group II was fed a KD. Group III animals were fed a basal diet + 5% low-MW (91 kDa) chitosan.

### Rat euthanasia and tissue preparation

After 45 days, rats were euthanized with an overdose of phenobarbital sodium salt, the neck skin was incised, and the SSGs were dissected carefully. The SSG was dissected for staining with hematoxylin and eosin (H&E) as a routine stain, alpha-smooth muscle actin (α-SMA) immunohistochemical marker as a widely characterized cytoskeletal protein that represents the hallmark of myofibroblast differentiation and Congo red special stain to detect the amyloid structure of protein aggregates. SSG specimens were fixed in 10% neutral-buffered formalin for one day and then dehydrated in ascending grades of alcohol, cleared in xylene, and embedded in soft paraffin.

For H&E, the dewaxed sections were rehydrated and then placed in Ehrlich’s hematoxylin for 30 min followed by 1% eosin Y for 10 min. For α-SMA, the sections were deparaffinized first and then rehydrated and washed three times with phosphate-buffered saline (PBS). The sections were incubated with the primary antibody (mouse monoclonal antibody to α-SMA, 1/500 dilution, clone 1A4, ab7817, Abcam) in a humid chamber overnight and then washed three times with PBS. Thereafter, these sections were incubated with the corresponding biotinylated peroxidase-conjugated secondary antibody for one hour and then rinsed three times in PBS. 3,3’-Diaminobenzidine (DAB)-hydrogen peroxide was used as a chromogen to stain α-SMA-bound structures and to localize the site of immunoreaction [23]. For Congo red, the sections were deparaffinized and washed with distilled water, and the specimens were dried for 5 min and defatted in acetone for 10 min. The specimens were immersed for 10–20 min in Congo red B stain solution diluted with Congo red A solution by 1:10 [24].

Slides were photographed using a digital camera (VE-MC5 5.0 MP) installed on an Olympus microscope with a magnification lens. The resulting images were analyzed on an Intel® core I7® computer using Fiji ImageJ (version 1.51r; NIH, Maryland, USA) software. To measure the percentage of the staining area, the color deconvolution plugin was used. Five random fields from each slide were analyzed.

### Statistical analysis

Quantitative data from α-SMA immunostaining and Congo red staining were statistically analyzed using one-way ANOVA with GraphPad Prism (version 8.0.0 for Windows, GraphPad Software, San Diego, CA, USA, www.graphpad.com, accessed on 21 April 2022), followed by Tukey’s multiple comparisons post hoc test. Data are presented as the mean ± standard deviation of the mean, and significance was considered at *p* < 0.05.

## Results

### Hematoxylin and eosin results

**Fig. 1:**
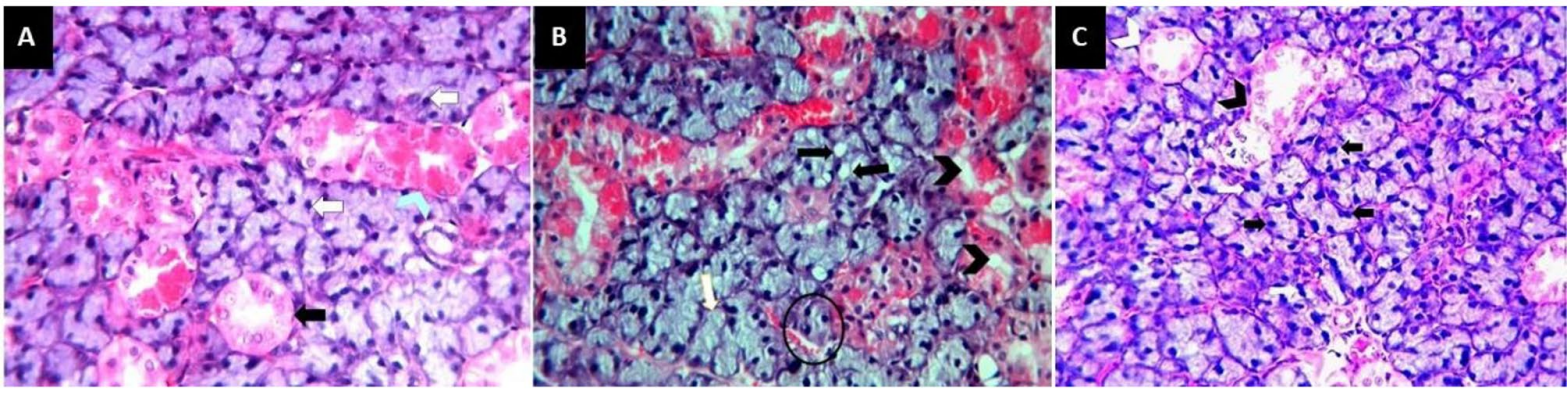
Soft paraffin sections showing (**A**) normal histological features of rats’ SSGs in group I with spherical serous acini without any mucous demilunes, well developed intercalated and striated ducts (**black arrow**), markedly developed GCTs (**blue arrow head**) and acinar cells have dark staining cytoplasm with normal basophilic nuclei (**white arrow**); (**B**) group II sections revealing disturbed architecture with a shrinkage of serous acini (**circle**), cytoplasmic vacuolization in both the acini (**black arrow**) and duct system (**black arrow head**) and some acinar cells have a deeply stained pyknotic nuclei (**white arrow**); (**C**) group III sections showing nearly the same histological features as group I with normal shape of serous acini, striated duct (**white arrow head**), granular convoluted tubules (**black arrow head**) and some acinar cells have binucleated nucleus (**white arrow**) and a dark basophilic staining cytoplasm (**black arrow**); H&E stain 400x

Group I section showed normal histological features of rat SSGs. The spherical serous secretory acini had no mucous demilunes. The acinar cells had a pyramidal shape with darkly stained cytoplasm and centrally situated basophilic rounded nuclei. The duct system was clearly seen in the form of well-developed intercalated and striated ducts with markedly developed granular convoluted tubules (GCTs). The GCTs consisted of pyramidal shaped cells with apically positioned acidophilic granules and basally situated large nuclei (Fig. 1a). Meanwhile, group II sections showed a disturbed architecture with shrinkage of the serous acini, and cytoplasmic vacuolization was clearly observed in both the acini and duct system. The acini and duct system showed severe destruction with a faintly stained basophilic cytoplasm and many deeply stained pyknotic nuclei (Fig. 1b). Group III sections revealed nearly the same histological features as group I, as the serous acini had a normal shape with darkly stained cytoplasm and rounded basally situated nuclei, with some acinar cells having a binucleated nucleus. The ducts as well as the GCTs were clearly demarcated (Fig. 1c).

### Immunohistochemical results of α SMA

**Fig. 2:**
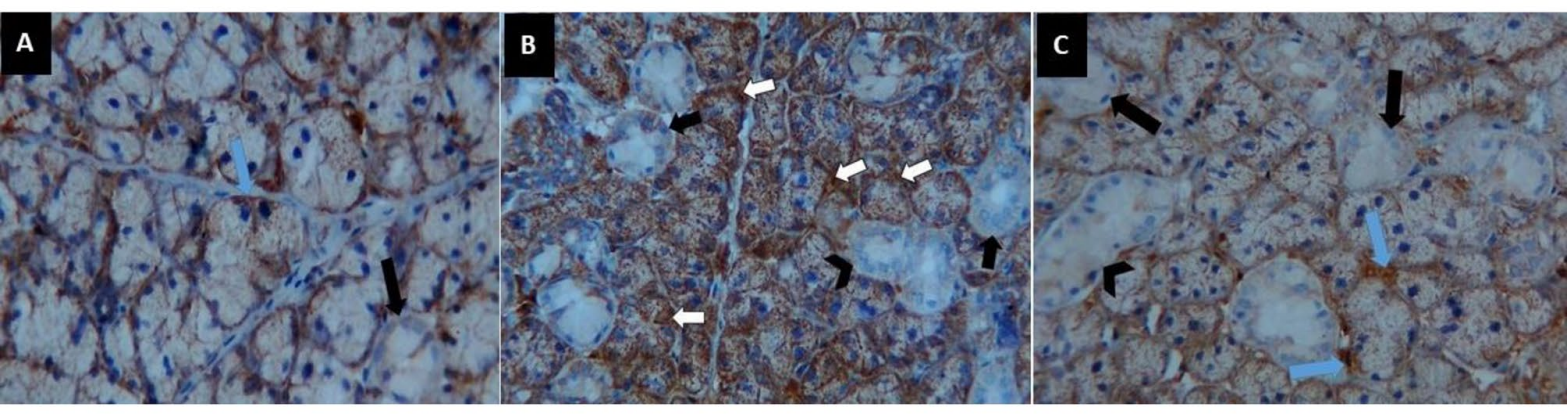
Soft paraffin sections showing (**A**) weak positive reaction of α- SMA to myoepithelial cells cytoplasm (**blue arrow**) and negative reaction to striated duct (**black arrow**) in group I, (**B**) group II sections showing very strong positive reaction of α- SMA to myoepithelial cells cytoplasm (**white arrow**) and negative reaction to striated ducts (**black arrow**) and GCTs (**black arrowhead**), (**C**) group III sections showing weak positive reaction of α- SMA to myoepithelial cells cytoplasm (**blue arrow**) and negative reaction to striated ducts (**black arrow**) and GCTs (**black arrow head**), α SMA immunohistochemistry stain 400x

In all groups, there is a significant difference between them (F ratio = 141.0 and *P* value < 0.001) by One-way ANOVA. Group II had the highest mean value (91.41 ± 3.30), while Group I had the lowest (56.13 ± 3.96). Group III had a mean value of 60.70 ± 3.55. Tukey’s multiple comparisons post hoc test revealed significant differences between groups I & II and II & III (*P* < 0.05). Meanwhile, there was a nonsignificant difference between groups I and III (*P* > 0.05) (Fig. 2) (Table 2).

### Results of congo red special stain

**Fig. 3:**
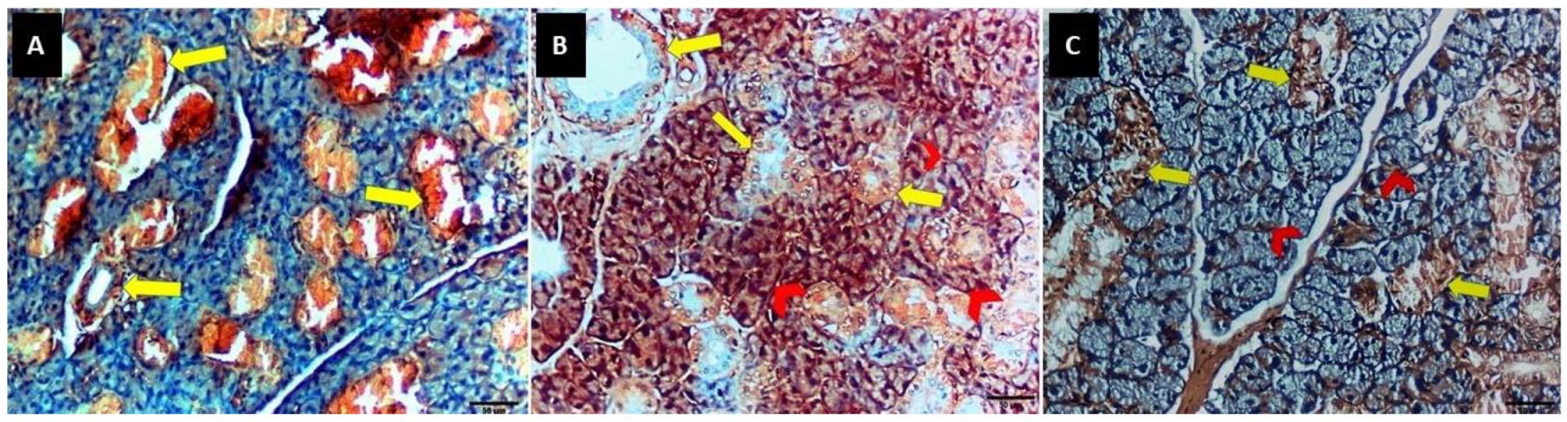
Soft paraffin sections showing (**A**) slight reaction at periductal area (**yellow arrow**) with no reaction to periacinar area in group I, (**B**) group II sections revealing severe reaction at periductal area (**yellow arrow**) and periacinar area (**red arrow head**), (**C**) group III sections showing slight reaction to periductal area (**yellow arrow**) and mild reaction to periacinar area (**red arrow head**), Congo red special stain 400x

In One-way ANOVA revealed a significant difference between the three groups (F ratio = 374.1 and *P* value < 0.001). Group II had the highest mean value (68.10 ± 5.04), while Group I had the lowest (16.87 ± 2.19). Group III had a mean value of 19.50 ± 1.78. Tukey’s multiple comparisons post hoc test revealed significant differences between groups I & II and II & III (*P* < 0.05). Meanwhile, there was a nonsignificant difference between groups I and III (*P* > 0.05) (Fig. 3) (Table 2).

**Table 2:** Descriptive statistics for SMA and Congo red staining in the different groups

Groups	**Mean ± SD**	
	**α SMA**	**Congo red**
I Control group	56.13 ± 3.96^A^	16.87 ± 2.19^A^
II Ketogenic diet group	91.41 ± 3.30^B^	68.10 ± 5.04^B^
III Chitosan group	60.70 ± 3.55^A^	19.50 ± 1.78^A^
ANOVA (F ratio, *P* value)	(141.0, < 0.001)	(374.1, < 0.0001) ^†^

^†^ANOVA test; different superscript alphabet (A, B) in the same column indicates statistical significance (Tukey’s HSD Post hoc test); results were considered significant when *P* < 0.05

## Discussion

The ketogenic diet began in 1920 as a treatment for childhood seizures; however, it was considered a starvation metabolic condition. KD involves high fat and low protein and carbohydrates, which change the metabolic state of the brain to metabolize the ketone bodies formed by the liver [25]. Currently, the KD changes from epileptic therapy to a traditional diet used for weight loss. However, it may have some side effects on long-term use [26]. In the present study, the main objective was to test the null hypothesis that there is no difference between the three groups against the alternative group. Therefore, the rats were divided randomly into three groups: the control group, which received a balanced diet; the KD group, which received a KD; the chitosan group, which received a balanced diet with dietary chitosan supplementation; and the mixed group, which received a KD concomitant with dietary chitosan supplementation, for 45 days.

The histological results of H&E staining for group II showed severe degenerative effects on the SSGs of rats, as there was abnormal architecture of the acini with severe vacuolization and some pyknotic nuclei in the acini, intercalated ducts, striated ducts and GCTs. In addition, α SMA immune stain was very strong on myoepithelial cells of acinar and intercalated duct cells, and this could also be attributed to its contractile function that helps to expel secretions from the Lumina of the secretory units and ducts. Additionally, it showed a severe Congo red stain reaction in the periacinar and periductal areas that could be attributed to SSG amyloidosis.

To the best of our knowledge, this is the first paper to study the histological effect of a keto diet on the structure of salivary glands. Therefore, our result is nearly in agreement with Tomečková et al. (2017) [26], who studied the effect of KD on human saliva compared to a low-carb diet and mixed them by atomic force microscopy. They found that the crystals in the KD group had a larger size, from 1000 to 2000 nm, and the highest surface roughness reached 84.9 nm, in comparison to the mixed diet, which showed crystal sizes from 400 to 1000 nm with an increased surface roughness of 25.2 nm. On the other hand, there were no crystals found in the group fed the low-carb diet.

Arsyad et al. (2020) [27] used a KD on rats for 60 days and observed weight loss and low blood sugar but also high metabolic acidosis and anemia and decreased plasma antioxidant enzyme levels. Therefore, interpreting the facts of the harmful effect may occur due to acidosis as a side effect of this diet type, which could affect salivary gland cells. However, it is a main concern during diet initiation and acute intercurrent illnesses [28].

In the chitosan group, the acini were deeply basophilic with basophilic, rounded nuclei. The intralobular ducts and GCTs showed a normal appearance and normal round nuclei with normal acidophilic staining. This group resembled the control group, especially in the immunohistochemistry reaction, which had a weak reaction to the SMA antibody. Likewise, the chitosan group stained by Congo red showed a mild reaction in the periacinar and periductal areas, which was not significant compared with the control group. Our result was in agreement with a study showing the protective effect of chitosan on SSGs against the effect of monosodium glutamate, which finally showed that chitosan had a prophylactic effect against cytotoxicity formed by monosodium glutamate [29].

However, Yang and Hsiao (2015) [18] showed that chitosan has the ability to promote tissue structure formation in the salivary gland by regulating components of the basement membrane. However, basement membrane component regulation concentrates the local environment that supports the structural formation of the salivary gland [30].

Finally, we should reconsider the effect of a KD on the health of humans. Furthermore, many practical experiments should be performed to achieve either poor or good effects of a KD on human health. As our experiment just takes the action of KD on SSG, we have limitations on other organs, and we need more experiments for the interpretation of the facts of the KD effect. Other experiments also can be done to see the effect of KD on the SSG of humans by more methods.

## Conclusion

Based on the results of this study, we conclude that KD has a deleterious effect on rats’ SSG, while a chitosan-based diet ameliorates these damaging effects. However, we used H&E staining, immunohistochemical stain, and special stain for amyloidosis, all of them gave the same harmful effect of KD to SSG.

## Data Availability

The datasets used and/or analyzed during the current study are available from the corresponding author upon reasonable request.

## Abbreviations

SSGs: submandibular salivary glands
KD: ketogenic diet
ARRIVE: Animal Research:Reporting of In Vivo Experiments
H&E: hematoxylin and eosin
α-SMA: alpha-smooth muscle actin
GAGs: glycosaminoglycans
ANOVA: analysis of variance
PBS: phosphate-buffered saline
DAB: 3, 3’diaminobenzidine
GCTs: granular convoluted tubules
